# Modulation of Central Carbon Metabolism by Acetylation of Isocitrate Lyase in *Mycobacterium tuberculosis*

**DOI:** 10.1038/srep44826

**Published:** 2017-03-21

**Authors:** Jing Bi, Yihong Wang, Heguo Yu, Xiaoyan Qian, Honghai Wang, Jun Liu, Xuelian Zhang

**Affiliations:** 1 State Key Laboratory of Genetic Engineering, School of Life Science, Fudan University, Shanghai, China; 2 NPFPC Key Laboratory of Contraceptives and Devices, Shanghai Institute of Planned Parenthood Research, Institutes of Reproduction and Development, Shanghai, China; 3 Department of Molecular Genetics, Faculty of Medicine, University of Toronto, Toronto, Ontario, Canada

## Abstract

Several enzymes involved in central carbon metabolism such as isocitrate lyase and phosphoenolpyruvate carboxykinase are key determinants of pathogenesis of *Mycobacterium tuberculosis (M. tb*). In this study, we found that lysine acetylation plays an important role in the modulation of central carbon metabolism in *M. tb*. Mutant of *M. tb* defective in sirtuin deacetylase exhibited improved growth in fatty acid-containing media. Global analysis of lysine acetylome of *M. tb* identified three acetylated lysine residues (K322, K331, and K392) of isocitrate lyase (ICL1). Using a genetically encoding system, we demonstrated that acetylation of K392 increased the enzyme activity of ICL1, whereas acetylation of K322 decreased its activity. Antibodies that specifically recognized acetyllysine at 392 and 322 of ICL1 were used to monitor the levels of ICL1 acetylation in *M. tb* cultures. The physiological significance of ICL1 acetylation was demonstrated by the observation that *M. tb* altered the levels of acetylated K392 in response to changes of carbon sources, and that acetylation of K392 affected the abundance of ICL1 protein. Our study has uncovered another regulatory mechanism of ICL1.

Tuberculosis (TB), caused by *Mycobacterium tuberculosis (M. tb*), continues to be a major global health problem, causing 1.5 million deaths and 9.6 million new infections in 2014. The success of *M. tb* as a bacterial pathogen is in part owed to its ability to adapt to changing environments and establish a long-term infection in the host. In the majority of individuals infected with *M. tb*, the bacteria establish a latent, asymptomatic infection that persists for decades^1^,^2^. An estimated one-third of the world’s population is latently infected with *M. tb*, representing a major disease reservoir and potential risk. The host environment that the bacteria encounters includes the acidic phagolysosomes (pH 5.8–6.2) in macrophages^3^, the nutrient depleted and hypoxic center of granulomas^4^,^5^. How *M. tb* survives these harsh conditions has been a subject of intensive research. Such knowledge may lead to the development of drugs specifically targeting the latent bacteria. Much of the work has focused on the transcriptional response of *M. tb* to various environmental stresses, including low pH, nutrient limitation, and hypoxia. Accordingly, sets of *M. tb* genes differentially expressed under these conditions have been identified, such as the DosR regulon that responds to hypoxia^6^,^7^,^8^,^9^ and low pH^10^.

The physiological consequence of the observed transcriptional response of *M. tb* remains largely unknown, which presumably enables the bacteria to alter or reduce metabolism, leading to latency. Supporting this, a growing body of evidence suggests that *M. tb* alters central carbon metabolism and uses host fatty acids rather than carbohydrates as the predominant carbon substrate during infection^5^. Even-chain-length fatty acids are metabolized by β-oxidation to acetyl-CoA, which is then assimilated by the glyoxylate shunt comprising of isocitrate lyase and malate synthase. The glyoxylate shunt bypasses the CO_2_-generating steps of the tricarboxylic acid (TCA) cycle, resulting in the net assimilation of carbon and replenishing of the pool of TCA cycle intermediates necessary for gluconeogenesis and other biosynthetic processes^11^. β-Oxidation of odd-chain-length fatty acids yields additional propionyl-CoA, which is assimilated via the methylcitrate cycle and requires the activity of methylcitrate lyase^12^. Mutants of *M. tb* lacking isocitrate and methylcitrate lyase did not grow on fatty acids *in vitro* and were defective in the persistent stage of infection in mice^12^,^13^,^14^. In addition, increased expression level of isocitrate lyase was observed in *M. tb* cultures grown under hypoxia^15^,^16^, at low pH^17^, after macrophage infection^18^, and in lung granulomas of TB patients^19^, supporting a critical role of isocitrate lyase in *M. tb* pathogenesis.

*N*^ɛ^-acetylation of lysine is one of the most frequently occurred and conserved post-translational modifications. Initially described in histone modification in eukaryotes^20^, lysine acetylation have been increasingly observed in non-nuclear proteins^21^,^22^ and in organisms ranging from bacteria to humans^23^,^24^. In bacteria, lysine acetylation is more prevalent than phosphorylation, and the majority of acetylated proteins are involved in central metabolism and translation, implying an important role in regulation of cellular processes^25^,^26^,^27^. In this study, we examined the role of lysine acetylation in the adaptation of *M. tb* to various environmental conditions. We found that lysine acetylation plays a critical role in regulation of central carbon metabolism in *M. tb*.

## Results

### Proteins involved in metabolism and translation are enriched in *M. tb* lysine acetylome

To identify acetylated proteins in *M. tb* H37Ra, we digested whole cell lysate with trypsin and then mixed it with an acetyllysine-specific antibody. The enriched peptides were separated and mapped by liquid chromatography coupled to tandem mass spectrometry (LC-MS/MS). A total of 441 unique acetylated peptides were identified in *M. tb* grown in 7H9 media under aerobic conditions, which were matched to 286 proteins (Supplementary Dataset S1). Of these, 75 proteins have more than one acetyllysine. Ge and co-workers identified 226 acetylated peptides in 137 proteins of *M. tb* H37Ra (herein referred as the GF dataset)^28^. Compared to our dataset, 72 common peptides in 45 proteins were identified and the overlap is highly significant (hypergeometric *p* = 1.4*e*^−19^). After combining these two datasets, 607 acetylated peptides in 355 proteins were identified in H37Ra. Xie *et al*. identified 1128 acetylated peptides in 658 proteins of *M. tb* H37Rv^29^. Since these two strains are closely related to each other and nearly identical, we also compared the two Ra datasets with the Rv dataset. The overlap between our Ra dataset and the Rv dataset is highly significant, with 152 acetylated peptides in 117 proteins were identified in both experiments (*p* = 6.8*e*^−25^). One hundred and four overlapped peptides in 73 proteins were found between the GF Ra and the Rv dataset (*p* = 1.1*e*^−23^). Comparing all three datasets, 33 proteins containing 43 overlapped peptides were identified. Discrepancies of bacterial acetylomes identified by mass spectrometry had previously been reported in other systems such as *E. coli*^25^,^30^,^31^. The difference in the number of acetylated peptides identified in three independent studies likely originates from differences in experimental conditions, including culture conditions and the affinity of antibody used for peptide enrichment. Nonetheless, acetylomes identified by the proteome-wide approach provide putative targets of acetylation, which can be validated by future studies^27^. By combining the three datasets, a total of 765 nonredundant acetylated proteins of *M. tb* were identified (Supplementary Dataset S2). We consider the combined dataset of the three separate studies representative of the lysine acetylome of *M. tb* identified thus far.

The *M. tb* genome was annotated to consist of 11 functional groups^32^. Of the 286 acetylated proteins identified in our experiment, 40, 83, and 44 proteins are involved in ‘lipid metabolism’ (14.0%), ‘intermediary metabolism/respiration’ (28.0%), and ‘information pathways’ (15.4%), respectively, which are in significantly higher proportions compared to the overall distribution of these proteins in the whole bacteria (Supplementary Fig. S1, the hypergeometric *p* value for identifying acetylated proteins in these categories were 1.1*e*^−7^, 0.004, and 5.1*e*^−10^, respectively). The probability of identifying acetylated proteins belonging to other functional groups (e.g. virulence, regulatory function) was the same as by a random process. A similar result was found when analyzing the other two datasets (GF Ra and Rv) or the combined dataset of three studies (Supplementary Fig. S1). Of the total 765 acetylated proteins of *M. tb*, those belonging to lipid metabolism, intermediary metabolism/respiration, and information pathways are significantly enriched, accounting for 12.8% (*p* = 9.0*e*^−18^), 30.8% (*p* = 3.7*e*^−12^), and 13.4% (*p* = 3.3*e*^−22^) of the lysine acetylome. Moreover, GO (gene ontology) enrichment analysis of the 765 proteins also revealed that proteins involved in metabolism (e.g., nucleotide and lipid metabolism) and translation are significantly enriched (Supplementary Fig. S1).

### *M. tb* altered lysine acetylome under hypoxic conditions

Given that a large number of acetylated proteins are involved in metabolism, it is of interest to determine if the lysine acetylome of *M. tb* changes under hypoxia, an *in vitro* condition that is most commonly used to mimic latent *M. tb* infection in the host. Therefore, we analyzed the acetylated proteins of H37Ra cultures grown under hypoxia, which has not been done previously^28^,^29^. Interestingly, only 111 acetylated peptides were identified from the hypoxic cultures, including 55 peptides that were also identified under aerobic conditions (Supplementary Dataset S1). The 56 acetylated peptides unique to the hypoxic cultures were mapped to 49 proteins, which is in much lower number than that identified under aerobic conditions. The differential pattern of protein acetylation may reflect differences of metabolic activity between aerobic and hypoxic cultures. This result also suggests that lysine acetylation is dynamically regulated and that *M. tb* undergoes rapid and reversible lysine acetylation to facilitate adaptation to the changing environment.

### *M. tb* lacking the sirtuin deacetylase exhibited altered carbon metabolism

**Figure 1:**
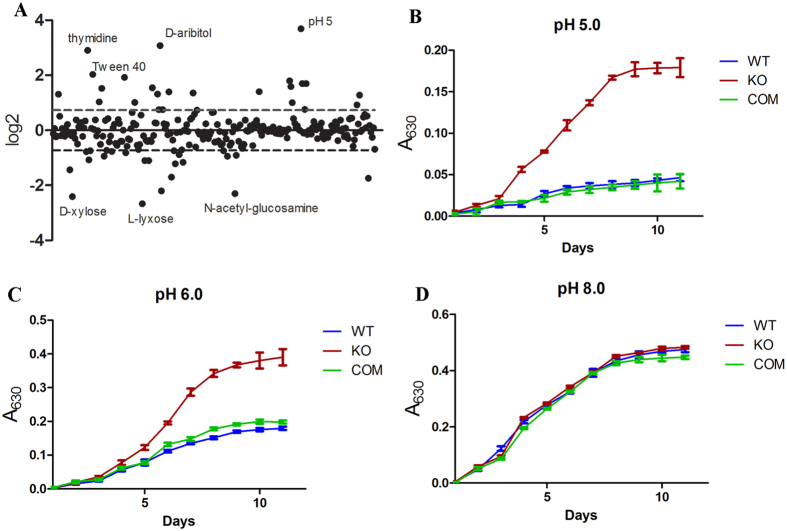
Growth phenotypes of Δ*npdA*. (**A**) Differential growth of Δ*npdA* using the BIOLOG Phenotype Microarray^TM^ plates. Data are from duplicate plates. The log_2_ ratio (Δ*npdA*/WT) was plotted and the standard deviation was plotted as the dashed lines. (**B–D**) Δ*npdA* exhibited better growth under acidic pH. KO: Δ*npdA*; COM: the complemented strain. Results are combined data (mean ± s.d.) from two independent experiments, and each experiment was done in triplicate.

Lysine acetylation is reversible, which in bacteria is carried out by the sirtuin family of NAD^+^-dependent deacetylases. MRA_1161 (Rv1151c, NpdA) is the only sirtuin homolog found in *M. tb* genome^27^. Previously, it was shown that deletion of *npdA* increased the level of lysine acetylation in *M. tb* H37Ra^28^. To determine the effect of lysine acetylation on the physiology of *M. tb*, we constructed an *npdA* knock out strain of H37Ra (Δ*npdA*) and compared its growth phenotype with the wild type (WT) strain under various conditions using the BIOLOG Phenotype Microarray^TM^ plates. A total of 96 × 16 conditions including 190 different carbon sources, 380 nitrogen sources, 59 phosphorus sources, 35 sulfur sources, 95 nutrient supplements, and pH conditions ranging from 3.5 to 9.5, were tested. WT and Δ*npdA* strains showed detectable growth in 251 media. Of these, Δ*npdA* showed endpoint growth increased ≥2 fold in 20 media and reduced ≤2 fold in 11 media compared to WT (Fig. 1A). Majority of the differences (19 conditions) were observed in the use of carbon sources, 3 differences in nitrogen sources and 7 differences in pH (Supplementary Table S1).

Δ*npdA* exhibited more growth than WT under acidic pH (Fig. 1A). To confirm this, we re-grew Δ*npdA*, WT, and the complemented strain in 7H9 media adjusted to different pH. Indeed, Δ*npdA* grew significantly better than WT or the complemented strain at pH 5.0 or 6.0, but not at pH 8.0 (Fig. 1B–D).

**Figure 2:**
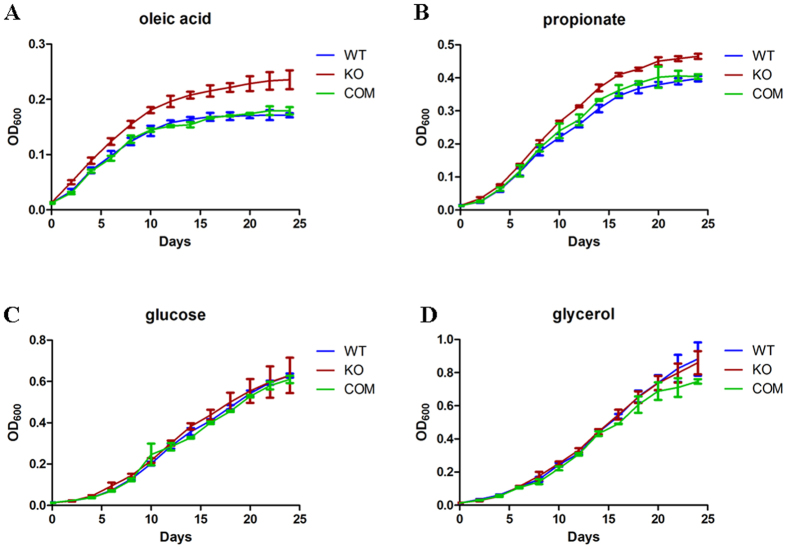
Δnp*dA* exhibited enhanced growth in fatty acids. WT, Δ*npdA* (KO) and the complemented strain (COM) were grown in 7H9-10%OADC-0.05% Tween-80 to mid-log phase (OD_600_ = 0.4–0.6), washed and resuspended in 7H9-0.05% tyloxapol. Aliquots (OD_600_ = 0.5) of the cultures were diluted 1:50 (v/v) into 100 ml 7H9-0.5% BSA-0.085% NaCl-0.05% tyloxapol supplemented with individual carbon sources (5 mM of each): glucose, glycerol, sodium acetate, sodium propionate or oleic acids. The cultures were grown at 37 °C for 24 days. Results are combined data (mean ± s.d.) from two independent experiments, and each experiment was done in triplicate. Statistically significant differences were found between Δ*npdA* and WT or between Δ*npdA* and the complemented strain when grown in oleic acids or propionate (**A** and **B**) as analyzed by two-way ANOVA (*p* < 0.05).

Δ*npdA* showed 4.1-fold more growth in Tween-40 than WT, and to a lesser extent in Tween-80 (1.3 fold). Tween-80 is routinely used in liquid media for dispersed growth of *M. tb*, which otherwise tends to form aggregates due to the lipid-rich cell surface. Tween-80 is unstable and breaks down to oleic acid, which can serve as a fatty acid carbon source for mycobacterial growth. The observation that Δ*npdA* grew better in Tween-80 suggests that it may grow better on oleic acid. To test this, we examined the growth of Δ*npdA* in 7H9 media containing oleic acid as the sole carbon source. Consistently, Δ*npdA* grew more rapidly and reached a higher biomass than WT or the complemented strain (Fig. 2A). Δ*npdA* also grew better when propionate was used as the sole carbon source (Fig. 2B). No significant differences were found between these two strains when they were grown in media using glucose, glycerol, or acetate as the sole carbon source (Fig. 2C and D). Comparing the growth in different carbon sources, WT exhibited the most rapidly growth in glycerol, followed by glucose, then acetate and propionate, and lastly oleic acid (Supplementary Fig. S2). A similar order was observed for Δ*npdA*, except that it exhibited similar growth rates in glucose and propionate (Supplementary Fig. S2). Taken together, these data suggest that Δ*npdA* has altered central carbon metabolism and increased its ability to grow on fatty acids.

### Acetylation of isocitrate lyase affects enzyme activity

*M. tb* employs the glyoxylate shunt and methylcitrate cycle to replenish the TCA cycle when fatty acids are used as the main carbon source^11^. Isocitrate lyase and methylcitrate lyase are key enzymes involved in the glyoxylate shunt^13^ and methylcitrate cycle^12^, respectively. In *M. tb* H37Rv or Ra, the dual activity of isocitrate lyase and methylcitrate lyase is carried out by a single protein (ICL1, MRA_0473, Rv0467)^33^. Some strains of *M. tb*, such as the Erdman strain, contain two copies of *icl* genes (*icl1* and *icl2*), but *icl2* in H37Rv or Ra contains a frameshift mutation and is therefore a pseudogene. We confirmed that the H37Ra strain used in the current study also contains the frameshift mutation in *icl2*.

We found that three lysine residues (K322, K331 and K392) of ICL1 of H37Ra were acetylated (Supplementary Dataset S1). To validate the ICL1 acetylation sites identified by the proteomic approach, we first determined if acetylation of K322, K331, and K392 affects its enzyme activity. The effects of lysine acetylation in a given protein have been routinely studied using recombinant mutants in which lysine residues are substituted with glutamine as a mimic of acetyllysine, or with arginine as a mimic of non-acetylated lysine. Accordingly, we generated site-specific mutants of ICL1 in which K322, K331 or K392 was substituted with arginine (R) or glutamine (Q) individually. These recombinant mutant proteins were expressed in *E. coli* BL21 and purified. These mutations did not affect the gross structure of ICL1 protein as analyzed by circular dichroism.

**Figure 3:**
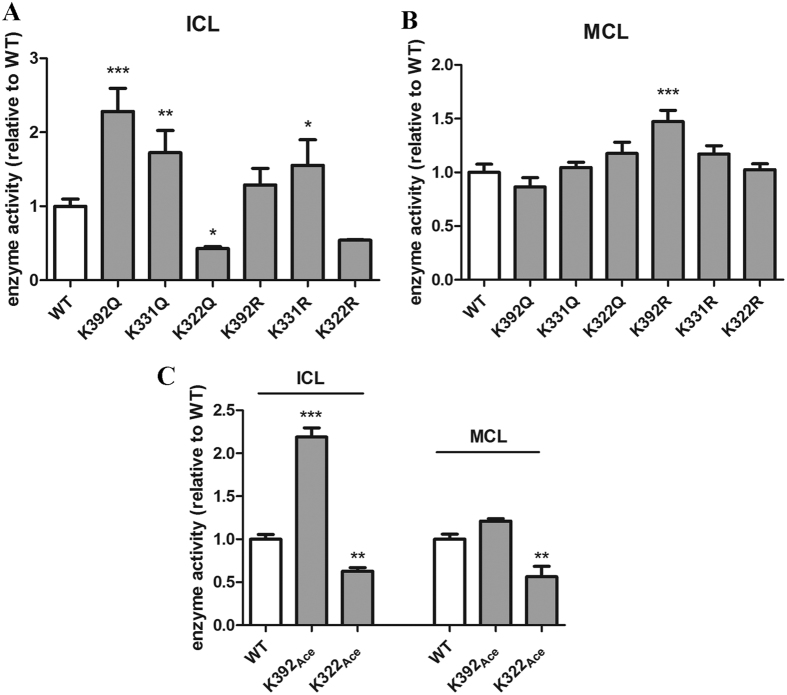
Effects of acetylation at K322 and K392 on ICL1 enzyme activities. (**A**) Isocitrate lyase (ICL) and methylcitrate lyase (**B**) activities of purified ICL1 mutant proteins. The data were normalized against the activity of WT protein and plotted as mean ± s.d. (*n* = 3). Statistical analysis was performed by one-way ANOVA comparing each mutant protein with WT. *p < 0.05; **p < 0.01; ***p < 0.001. (**C**) Isocitrate lyase (ICL) and methylcitrate lyase (MCL) activities of purified ICL1 proteins in which K322 or K392 was acetylated. The data were normalized against the activity of WT protein and plotted as mean ± s.d. (*n* = 3). Statistical analysis was performed by Student’s *t-*test comparing each mutant protein with WT. **p < 0.01; ***p < 0.001.

The enzyme activities (including both isocitrate lyase and methylcitrate lyase activities) of ICL1 mutant proteins were analyzed. Interestingly, mutations of these residues had distinctive effects on isocitrate lyase (ICL) activity (Fig. 3A). The substitution of K392 with glutamine (K392Q) caused a large increase in ICL activity, reaching ~2.3 fold of WT enzyme. K331Q also increased enzyme activity but to a lesser extent (~70%). By contrast, K322Q exhibited a decreased ICL activity, which was ~43% of WT enzyme. The substitution of K392 or K322 with arginine did not have a significant effect, and the K331R substitution increased the activity by 50%.

The substitution of these three residues with glutamine or arginine did not significantly change the methylcitrate lyase (MCL) activity, with the exception of the K392R substitution, which exhibited ~50% increase in activity (Fig. 3B).

To confirm the effect of acetylation on the enzyme activity of ICL1, we employed a system of genetically encoding *N*^*ε*^ -acetyllysine to prepare recombinant ICL1 protein in *E. coli*^34^. This system allows acetylation at specific lysine residue of a given protein due to the suppression of lysine TAG stop codon by the *N*^*ε*^-acetyllysine-conjugated amber suppressor tRNA, which results in homogeneous protein containing acetyllysine at desired sites. We focused on K392 and K322, since substitution of these two residues with glutamine had the most drastic effect on the enzyme activity of ICL1. The acetylated proteins were expressed and purified from *E. coli* and analyzed for isocitrate and methylcitrate lyase activities.

The isocitrate lyase activity of K392-acetylated (K392_Ace_) and K322-acetylated (K322_Ace_) ICL1 was 2.2- and 0.6-fold of the WT enzyme, respectively (Fig. 3C). These results were consistent with the data of K392Q and K322Q (Fig. 3A). For methylcitrate lyase activity, acetylation at K392 had no significant effect. However, acetylation of K322 reduced the activity by 44%. These results indicate that acetylation of K392 in ICL1 enhanced isocitrate lyase activity but did not affect methylcitrate lyase activity, whereas acetylation of K322 reduced both isocitrate and methylcitrate lyase activities.

### Dynamic changes of ICL1 acetylation in *M. tb* grown in different carbon sources

**Figure 4:**
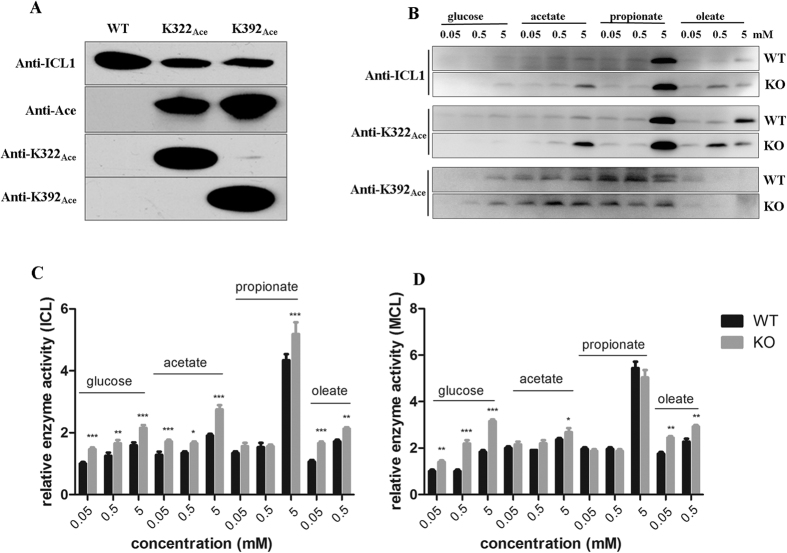
Expression and acetylation of ICL1 in *M. tb* under different carbon sources. (**A**) Western blot analysis of purified WT or ICL1 proteins in which K322 or K392 was acetylated. Equal amount of purified proteins (1 μg per lane) was analyzed by Western blot. Antibodies raised against ICL1 (anti-ICL1), K322 or K392 acetylated peptides (anti-K322_Ace_, anti-K392_Ace_), or acetyllysine (anti-Ace) were used. (**B**) Western blot analysis of cell extracts of WT and Δ*npdA* (KO) grown in different carbon sources at various concentrations. The cultures were grown in 7H9 media supplemented with 0.5% BSA, 0.085% NaCl and different carbon source at indicated concentration at 37 °C for 8 days. Cell extracts (20 μg per lane) was analyzed by Western blot with appropriate antibodies. Duplicate SDS-PAGE gel stained with Coomassie blue was used as the loading control (Supplementary Fig. S4A and B). Results are representative of three independent experiments. (**C**) Isocitrate lyase (ICL) activities of *M. tb* cultures grown in different carbon sources at various concentrations. The cell extracts of WT and Δ*npdA* (KO) grown in different carbon sources at indicated concentrations were analyzed. The data were normalized against the ICL activity of WT cultures grown in 0.05 mM glucose. (**D**) Methylcitrate lyase (MCL) activities of *M. tb* cultures grown in different carbon sources at various concentrations. The cell extracts of WT and Δ*npdA* (KO) grown in different carbon sources at indicated concentrations were analyzed. The data were normalized against the MCL activity of WT cultures grown in 0.05 mM glucose. For both (**C**) and (**D**), the data are plotted as mean ± s.d. (*n* = 3). Two-way ANOVA was performed to compare WT and Δ*npdA* at each condition. *p < 0.05; **p < 0.01; ***p < 0.001.

The above *in vitro* biochemical analysis demonstrated that acetylation of K322 and K392 of ICL1 had significant effects on its enzyme activities. To determine the physiological relevance of these modifications, we generated antibodies that specifically recognized acetyllysine at 392 and 322 of ICL1, respectively, and then used these antibodies to monitor ICL1 acetylation in *M. tb* cultures. These two antibodies demonstrated excellent specificity; the antibody raised against K392_Ace_ did not react with K322_Ace_ or WT protein, and conversely, the antibody that recognized K322_Ace_ did not bind K392_Ace_ or WT enzyme (Fig. 4A). Antibody raised against WT ICL1 protein (anti-ICL1) appeared to have a lower affinity for K322_Ace_ or K392_Ace_ protein (Fig. 4A).

To analyze the level of ICL1 containing K322_Ace_ or K392_Ace_ in *M. tb* cultures grown in different carbon sources, cells were first grown in 7H9 media supplemented with 10% OADC and 0.05% Tween-80 to log phase and washed with PBS, and aliquots of cells were then subcultured in 7H9 media containing 0.5% BSA, 0.085% NaCl and different carbon source at three concentrations (0.05, 0.5, 5 mM) for 8 days.

In cell extracts of WT bacteria grown in glucose, the protein level of ICL1 was low and was only detected in cultures grown at a high concentration of glucose (5 mM) using an anti-ICL1 antibody (Fig. 4B). The level of ICL1 increased in cultures grown in fatty acids compared to those grown in glucose at the same concentration, and was the highest in cultures grown in 5 mM propionate among the conditions tested. A smaller increase of ICL1 level was observed in cultures grown in acetate or oleic acid. A similar trend was observed for the Δ*npdA* strain grown in different carbon sources.

The pattern of ICL1 containing K322_Ace_ in both WT and Δ*npdA* grown in different carbon sources nearly mirrored that of the total ICL1 protein, thus higher levels of K322_Ace_-containing ICL1 were detected in cells grown in fatty acids than in glucose. Cultures grown in propionate exhibited the highest level of K322_Ace_-containing ICL1 protein in both WT and Δ*npdA* (Fig. 4B).

The pattern of K392_Ace_-containing ICL1 was different to that of the total ICL1 protein. The level of K392_Ace_-containing ICL1 was relatively stable and did not change significantly in cultures grown in acetate or propionate at different concentrations (0.05, 0.5, 5 mM) (Fig. 4B).

In a parallel experiment, the enzyme activities (isocitrate and methylcitrate lyase) of cell extracts from cultures grown in different carbon sources were measured (Fig. 4C). The isocitrate lyase activity in WT and Δ*npdA* grown in 5 mM propionate increased 1.7- and 1.4- fold, respectively, compared to that grown in 5 mM glucose. A smaller but reproducible increase was seen in cultures grown in acetate (Fig. 4C). Cultures grown in oleic acid had a similar isocitrate lyase activity to that grown in glucose. Both WT and Δ*npdA* grew poorly in oleic acid (Supplementary Fig. S2), and this may explain the low level of ICL1 protein and isocitrate lyase activity. A similar pattern was observed for the methylcitrate lyase activity (Fig. 4D).

Compared to WT, Δ*npdA* exhibited a general trend of higher isocitrate lyase activity, ranging from 1.2- to 1.6-fold of WT. A similar trend was observed for methylcitrate lyase activity, although the differences of methylcitrate lyase activity between these two strains were smaller. These results are in a general agreement with the observation that Δ*npdA* grew better in oleate- or propionate- containing media (Fig. 2).

### Acetylation of K392 affects the protein abundance of ICL1

**Figure 5:**
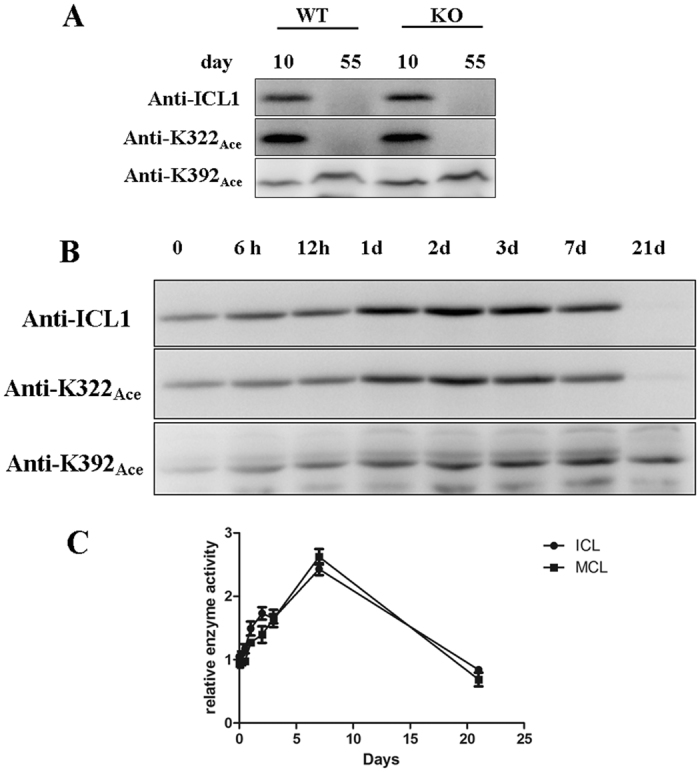
Expression and acetylation of ICL1 in *M. tb* cultures at different growth phase. (**A**) Expression and acetylation of ICL1 at different growth. WT and Δ*npdA* (KO) grown in 7H9 media supplemented with 10% OADC and 0.2% glycerol, and cultures at day 10 and day 55 of growth were collected and analyzed by Western blot. The loading control is shown in Supplementary Fig. S4C. (**B**) Expression and acetylation of ICL1 at different growth time. WT stain of *M. tb* H37Ra were grown in 7H9 media supplemented with 0.5% BSA, 0.085% NaCl and 5 mM propionate for 21 days. Cell extracts of cultures collected at indicated time points were prepared and analyzed by Western blot. The loading control is shown in Supplementary Fig. S4D. Results are representative of three independent experiments. (**C**) Enzymatic activities of ICL1. Isocitrate lyase (**C**) and methylcitrate lyase (**D**) activities from WT culture grown in 5 mM propionate at different time points were analyzed. The data are normalized against the activity of WT culture at time 0 and are plotted as mean ± s.d. (*n* = 3).

Western blot analysis revealed that the levels of total ICL1 and K322_Ace_-containing ICL1 decreased significantly in cultures grown to the extended stationary phase (day 55) compared to that grown in log phase (day 10), and this observation was seen in both WT and Δ*npdA* strains (Fig. 5A). By contrast, the level of K392_Ace_-containing ICL1 remained unchanged during the same growth period, suggesting that the unacetylated- or K322_Ace_-containing ICL1 protein were likely more susceptible to degradation. The anti-ICL1 antibody had a lower affinity to K392_Ace_-containing ICL1 (Fig. 4A) therefore could not detect the K392_Ace_-containing population at day 55. To examine this more closely, we re-grew the cultures in 5 mM propionate and monitored the changes of ICL1 at different time points up to 21 days (Fig. 5B). There was a gradual increase of total ICL1 protein which reached the highest level at early- to mid- log phase (2–7 days) of growth. Consistent with the above data, ICL1 protein could not be detected at day 21 of growth under the same experimental conditions. K322_Ace_-containing ICL1 exhibited a nearly identical pattern and was not detected at day 21. By contrast, high levels of K392_Ace_-containing ICL1 remained at day 21. The kinetics of ICL1 protein including its acetylated populations correlate with its enzyme activities (Fig. 5C). Thus, cultures grown in log phase exhibited higher levels overall of isocitrate and methylcitrate lyase activities than in stationary phase. A similar result was found for the Δ*npdA* strain (Supplementary Fig. S3). These data suggest that acetylation of K392 affects the abundance of ICL1.

## Discussion

Growing evidence has implicated central carbon metabolism, which includes glycolysis, gluconeogenesis, the pentose phosphate pathway, the glyoxylate shunt and the TCA cycle, as a key determinant of *M. tb* pathogenesis^11^. For examples, mutants of *M. tb* lacking enzymes in central carbon metabolism such as isocitrate lyase and phosphoenolpyruvate carboxykinase were severely attenuated in animal models of infection^14^,^35^. In addition, *M. tb* may have specifically evolved to optimize the carbon flux across a diverse range of environmental conditions^36^. Unlike many other bacteria, which use the carbon catabolite repression system to allow growth on preferred carbon substrate from a mixture of different carbon sources, *M. tb* appeared to be able to catabolize multiple carbon sources simultaneously^36^. Understanding how *M. tb* modulates central carbon metabolism in response to fluctuation of nutrients may lead to new strategy for its control.

In this study, we have identified a previously unrecognized mechanism which regulates the enzyme activity of ICL1. We showed that acetylation of ICL1 affects its enzyme activity, which is dependent upon the specific residues that are modified. Acetylation of K392 of ICL1 not only increased its enzyme activity (2.2-fold), but also appeared to affect the abundance of ICL1 protein. Transcriptional regulation of ICL1 has previously been described. A 2.5-fold induction of *M. tb icl1* transcript was observed upon the exposure of *M. tb* cultures to acidic conditions (pH 5.5), and 3.5-fold induction was reported in the early stage of hypoxic cultures^16^,^17^. Combined with our data, these studies suggest that *M. tb* regulates the activity of ICL1 in response to changes of environmental conditions by at least two mechanisms: transcriptional regulation and post-translational modification via lysine acetylation. The stoichiometry of acetylation at K322 and K392 of ICL1 is currently unknown. The ability to acetylate specific lysine residues (K322 and K392) of ICL1, coupled with their differential effect on the enzyme activity, adds another layer of regulation that *M. tb* could employ, which may allow the bacteria to fine tune ICL1 enzyme activities and achieve the optimal growth under specific environmental conditions.

The biological significance of acetylation of ICL1 in *M. tb* was demonstrated in several ways. Firstly, acetylation of ICL1 can increase or decrease its enzyme activity depending on the specific residues that are modified. Substitution of K322 or K392 with glutamine, which mimics acetyllysine, resulted in a significant decrease or increase of isocitrate lyase activity, respectively, but had little effect on methylcitrate lyase activity (Fig. 3A and B). However, since this is only an approximation, the contribution of acetylation at these residues could not be precisely defined. To solve this problem, we used a genetically encoding system which allows the incorporation of *N*^*ε*^-ac etyllysine at K322 or K392 and generated homogeneous recombinant proteins. Studies of these homogeneously acetylated ICL1 proteins demonstrated unequivocally that acetylation of K322 reduced both isocitrate and methylcitrate lyase activities of ICL1, whereas acetylation of K392 increased isocitrate lyase activity but had little effect on methylcitrate lyase activity (Fig. 3C). Secondly, we found that *M. tb* altered the level of K392_Ace_-containing ICL1 in response to changes of carbon sources in growth media. Increased levels of K392_Ace_-containing ICL1 was observed in *M. tb* cultures grown in propionate- or acetate-containing media, compared to cultures grown in glucose at the same concentration (Fig. 4B). Thirdly, we found that acetylation of K392 of ICL1 appeared to affect the abundance of the protein (Fig. 5).

We found that the disruption of NpdA, the only annotated sirtuin family of deacetylases in *M. tb*, had a profound effect on the growth phenotype of *M. tb*. The Δ*npdA* strain exhibited greater growth rate and biomass yield in propionate- or oleic acid-containing media than WT strain. While it is difficult to attribute the growth phenotype by modification of a single enzyme, this could be explained, at least partially, by a general trend of higher isocitrate lyase and methylcitrate lyase activities in Δ*npdA* than in WT (Fig. 4C and D). The Δ*npdA* strain also exhibited enhanced growth under acidic conditions. Interestingly, a recent study demonstrated the effect of acidic pH on remodeling of the central metabolism pathways in *M. tb*^37^. As such, the two growth phenotypes of the Δ*npdA* strain, *i.e.*, the enhanced growth in fatty acid-containing media and improved growth under acidic conditions, may be connected.

In addition to ICL1, our proteomic analysis also identified two lysine residues (K122, K213) of isocitrate dehydrogenase (ICD2) that were acetylated. Control of carbon flux between the glyoxylate shunt and the TCA cycle can be achieved by coordinating the activity of isocitrate lyase and isocitrate dehydrogenase, which compete for a common substrate (isocitrate). This has been demonstrated in *E. coli*, in which that phosphorylation of isocitrate dehydrogenase, which inactivated its activity, directed the carbon flux towards the glyoxylate shunt instead of TCA cycle^38^,^39^. In *M. tb* genome, isocitrate dehydrogenase is encoded by two genes, *icd*1 and *icd*2. Like ICL1, acetylation of the two lysine resides of ICD2 may profoundly affect its enzyme activity. Future studies to determine the effect of lysine acetylation on ICD2 activity, and the effect of *npdA* deletion on ICD2 acetylation, together with our current finding on ICL1, may help to explain the preference of the Δ*npdA* strain for fatty acids for its growth.

## Materials and Methods

### Bacterial strains

*M. tb* H37Ra and its *npdA* deletion mutant (Δ*npdA*) were used in this study. *E. coli* DH5α was used for genetic manipulation of DNA and *E. coli* BL21 (DE3) was used for expression of recombinant ICL1 proteins.

### Proteomic analysis of acetylated proteins of *M. tb*

*M. tb* H37Ra was grown in Middlebrook 7H9 (Difco) broth supplemented with 0.5% bovine albumin fraction V, 0.085% NaCl, 0.0004% catalase, 0.2% glucose, and 0.2% glycerol at 37 °C with shaking to mid-log phase (OD_600_ = 0.3–0.5). Hypoxic cultures were prepared according to the Wayne model^40^. Briefly, *M. tb* H37Ra (OD_600_ = 0.1) was incubated in 7H9 medium in 25 ml screw-cap bottles with the headspace to medium ratio of 0.5. The sealed cultures were stirred at 60 RPM for 3 days to deplete oxygen, and then kept static for 3 weeks at 37 °C. Cultures were pelleted and stored at −80 °C. To prepare extracts, cells were washed twice with 100 mM Na_2_HPO_4_ pH7.5, 150 mM NaCl and resuspended in 10 mM Tris-HCl pH 7.5, 10 mM KCl, 10 mM nicotinamide and then disrupted by sonication. Nicotinamide was added to inhibit the activity of deacetylase. Soluble proteins were collected after centrifugation at 20,000 × g for 40 min at 4 °C and added with 5 mM DTT and incubated at room temperature for 10 min. Freshly prepared 15 mM iodoacetamide was then added and incubated in dark for 30 min at room temperature. The reaction was stopped by adding 15 mM cysteine and incubating at room temperature for 30 min. Proteins were precipitated with 85% ice-cold acetone and washed twice with 80% ice-cold acetone and vacuum dried. Proteins were dissolved in 50 mM ammonium bicarbonate pH 8.0 and concentration determined by the Bradford assay (Bio-Rad, Hercules, CA).

Protein digestion and peptide enrichment and identification were performed using methods described previously^41^. Briefly, proteins (5 mg) were digested with sequencing-grade trypsin at 1:50 ratio (trypsin/protein) at 37 °C overnight, and further digested with a trypsin/protein ratio of 1:100 for 3 h to allow complete digestion. Trypsin was inactivated by heating at 99 °C for 5 min. The supernatant obtained after centrifugation was vacuum-dried and re-dissolved in deionized water. The vacuum-drying and re-dissolving steps were repeated 4× to remove salt. The pellet was re-suspended in 50 mM Tris-HCl, pH 8.0, 100 mM NaCl, 1 mM EDTA, and 0.5% NP-40 and incubated with 200 μl Sepharose 4B beads conjugated with pan-anti-acetyllysine antibody with gentle agitation for 6 h at 4 °C (the generation of pan-anti-acetyllysine antibody was described previously^41^). The supernatant was removed, and the beads were washed with PBS 3× and rinsed with deionized water. Enriched peptides were eluted with 0.1% trifluroacetic acid buffer containing 5% acetonitrile. The resulting peptides were separated by nanoflow liquid chromatography, and analyzed by Q Exactive mass spectrometer via a nanoelectrospray ion source (Thermo Fisher Scientific). The acquization of mass spectra and data analyses were described previously^41^.

### Generation of the Δ*npdA* and complemented strains

The Δ*npdA* strain was constructed using the specialized transducing phage system^42^. Briefly, the upstream left arm flanking the *npdA (MRA_1161*) gene was PCR amplified using primers 5′-TTTTTTTTCCATAAATTGGGGGACCTGCCCGCTGAGTTCGG-3′ and 5′TTTTTTTTCCATTTCTTGGGCCCGCCCTGCTGAAATAGTCG3′, and the downstream right arm was amplified using primers 5′-TTTTTTTTCCATAGATTGGGAGATCCCCGCGC CGCTGAG-3′ and 5′-TTTTTTTTCCATAGATTGGGAGATCCCCGCGCCGCTGAG3′. The PCR products were digested with Van91I and ligated to compatible fragments of the counter-selectable vector p0004S^42^. The allelic exchange plasmids were then digested with PacI and ligated to vector phAE159^42^ pretreated with PacI. The ligation product was packaged using MaxPlax^TM^ Lambda Packaging Extracts (Epicentre Biotechnologies, Madison, WI) and the resulting phage was used to infect *E. coli* HB101. The phasmid DNA was transfected into *M. smegmatis* mc^2^–155 complement cells and propagated at 30 °C. The resulting phasmid was used to transfect *M. tb* H37Ra and cultures plated incubated on 7H11 plate containing hygromycin (75 μg/ml) at 37 °C. Hygromycin resistant clones were isolated and analyzed by PCR and DNA sequencing to confirm the deletion of *npdA*. To complement the mutant strain, the *npdA* gene was amplified using PCR primers 5′-TAAGAATTCATGCGAGTGGCGGTGCTCAG-3′ and 5′-CTTAAGCTTCTATTTCAGCAGGGCGGGC-3′, digested with EcoRI and HindIII and then ligated into shuttle vector pMV361. The resulting plasmid was transformed into the Δ*npdA* strain and plated on 7H11 plate containing kanamycin at 25 μg/ml.

### Phenotype microarray analysis

Phenotype microarray experiments were carried out using the BIOLOG Phenotype Microarray^TM^ plates (Biolog Inc. Hayward, CA) following the manufacture’s protocol. To prepare the inoculum for PM plates, the WT and Δ*npdA* strain of *M. tb* H37Ra were grown in 7H9-10%OADC-0.05% Tween-80 to mid-log phase (OD_600_ = 0.4–0.6), collected by centrifugation and then washed twice with 20 mM PBS (pH 6.8)-0.025% tyloxapol. The bacteria were incubated in IF-0A GN/GP (Biolog Inc. Hayward, CA) for 24 h at 25 °C as a starvation step and then adjusted to OD_600_ = 0.6. The inoculation solutions for each plate were prepared according to the manufacturer’s instruction (Biolog Inc. Hayward, CA). Aliquots of 100 μl bacterial cultures (OD_600_ = 0.04) were added each well of the PM plates. After plate inoculation, each plate was sealed with parafilm and incubated at 37 °C for 7 days. Metabolite utilization was measured as color changes of redox dye G using an ELISA reader at 630 nm. Tests were performed in duplicated and each test well was then compared with the negative control well of the respective PM plate.

### *M. tb* growth at different pH

The WT, Δ*npdA* and the complemented strain of *M. tuberculosis* H37Ra were grown in 7H9-10%OADC-0.05% Tween-80 to mid-log phase (OD_600_ = 0.4–0.6), collected by centrifugation and then washed twice with 20 mM PBS (pH 6.8). The bacteria (OD_600_ = 0.04) were then resuspended in 7H9-10%ADC media that were adjusted to different pH (pH5.0, pH6.0, pH8.0) and contained redox dye G. The cultures were incubated at 37 °C for 10 days. Metabolite utilization at 37 °C was measured as color changes of redox dye G using an ELISA reader at 630 nm every day. Two independent experiments were performed and each in triplicate.

### *M. tb* growth at different carbon sources

The WT, Δ*npdA* and the complemented strain of *M. tuberculosis* H37Ra were grown in 7H9-10%OADC-0.05% tween80 to mid-log phase (OD_600_ = 0.4–0.6), collected by centrifugation and then were washed twice with and resuspended in 7H9-0.05% tyloxapol. Aliquots (OD_600_ = 0.5) of the cultures were diluted 1:50 (v/v) into 100 ml 7H9-0.5% BSA-0.085% NaCl-0.05% tyloxapol supplemented with individual carbon sources (5 mM of each): glucose, glycerol, sodium acetate, sodium propionate or oleic acids. The cultures were grown at 37 °C for 24 days. Two independent experiments were performed, and each in triplicate.

### Expression and purification of ICL1 site-directed mutants

The *icl1* gene was firstly cloned into pET28a using primers 5′-GCCGGATCCATGTCTGTCGTCGGCACCC-3′ and 5′-GCCAAGCTTCTAGTGGAACTGGCCCTC-3′. The resulting plasmid was used as the template for site-directed mutagenesis, which was carried out using the KOD plus mutagenesis Kit (Thermo Scientific™) with appropriate primers. The point mutations were confirmed by DNA sequencing. The resulting expression constructs were individually transformed into *Escherichia coli* strain BL21 (DE3) cells and expression of ICL1 proteins were induced with 0.5 mM isopropyl-β-D-1-thiogalactopyranoside (IPTG). The proteins were purified by Ni^2+^ affinity chromatography (Qiagen) following the manufacturer’s instruction.

### Generation of homogenous ICL1 protein containing *N* ^ɛ^-acetyllysine at defined sites

To generate recombinant ICL1 protein that homogenously contains acetylated K322 or acetylated K392, we used the genetic engineering system developed by Chin and co-workers, which allows incorporation of acetyllysine into proteins expressed in *E. coli*^34^,^43^. This system uses the *N*^ɛ^-acetyllysyl-tRNA synthetase/tRNA_CUA_ pair of *Methanosarcina barkeri* to direct the site-specific incorporation of *N*^ɛ^-acetyllysine to the amber codon^34^. We cloned wild-type *icl1* into pET21a using primers 5′-AAACATATGATGTCTGTCGTCGGCACCCCGAAGAG-3′ and 5′-TAAAAGCTTGTGGAACTGGCCCTCTTCGGTGGAAC-3′, then replaced the codon for lysine at 322 or 392 (AAG) with an amber codon (TAG) by site-directed mutagenesis. The resulting plasmids and two other plasmids (pAcKRS-3 and pPylT)^34^ were transformed into in *E.coli* BL21 and plated on agar plates containing spectinomycin (50 μg/ml), kanamycin (50 μg/ml l), and ampicillin (150 μg/ml). Recombinant strains of *E. coli* containing the three plasmids were grown in LB media supplemented with the same antibiotics to mid-log phage (OD_600_ = 0.6), the culture was then added with 0.5 mM IPTG, 2 mM *N*-acetyllysine and 10 mM nicotinamide and incubated at 30 °C for 6 h. ICL1 proteins containing acetylated K322 or K392 were purified by Ni^2+^ affinity chromatography (Qiagen) following the manufacturer’s instruction.

### Assays for isocitrate lyase and methylcitrate lyase activities

Isocitrate lyase (ICL) and methylcitrate lyase (MCL) activities were assayed under similar conditions using a protocol previously described^15^,^33^. Each reaction mixture (200 μl) contained 50 mM MOPS pH 6.8, 5 mM MgCl_2_, 0.1 mM NADH, 7 units of lactate dehydrogenase and purified ICL1 protein (0.8 μg) or cell-free extract (50 μg). The mixtures were pre-incubated for 5 min at room temperature and the reaction initiated by adding either isocitrate (1 mM) or 2-methylisocitrate (1 mM) and monitored for change of absorbance at 340 nm.

### Preparation of antisera

To generate anti-ICL1 antibody, ICL1 protein was expressed and purified from *E. coli* BL21 harboring pET28a-*icl1* as described above and used to immunize rabbits for the production of polyclonal antibody.

To generate anti-K322_Ace_ and anti-K392_Ace_ antibodies, peptides containing acetylated lysine at 322 and 392, KLH-CPSFNWKK_Ace_HLDDAT and KLH-RGYTATK_Ace_HQREVG, respectively, were synthesized and used to immunize rabbits. Rabbits were repeatedly immunized eight times by each peptide during the two-month period, and the antisera were collected. The control peptides, CPSFNWKKHLDDAT and RGYTATKHQREVG, were used to remove non-specific antibodies from the anti-K322_Ace_ and anti-K392_Ace_ antisera, respectively.

### Western blot analysis

Standard Western blot procedures were used in this study. Purified recombinant protein (2 μg) or cell extracts (50 μg) were separated on 12% SDS-PAGE and then transferred PVDF membrane. The concentrations of primary antibodies used in the corresponding blot were anti-ICL1 (1:10000), anti-K322_Ace_ (1:10000), anti-K392_Ace_ (1:10000) or anti-acetyllysine monoclonal antibody (1:5000).

## Additional Information

**How to cite this article:** Bi, J. *et al*. Modulation of Central Carbon Metabolism by Acetylation of Isocitrate Lyase in *Mycobacterium tuberculosis. Sci. Rep.* **7**, 44826; doi: 10.1038/srep44826 (2017).

**Publisher's note:** Springer Nature remains neutral with regard to jurisdictional claims in published maps and institutional affiliations.

## Supplementary Material

Supplementary Materials

Supplementary Dataset S1

Supplementary Dataset S2
