# Supplementary material for: Modulation of Central Carbon Metabolism by Acetylation of Isocitrate Lyase in Mycobacterium tuberculosis
Source: Sci Rep. 2017 Mar 21;7:44826. doi: 10.1038/srep44826 (PMC5359664; doi:10.1038/srep44826)
Supplement: Supplementary Materials [file srep44826-s1.pdf]

Modulation of Central Carbon Metabolism by Acetylation of Isocitrate Lyase in *Mycobacterium tuberculosis*

Jing Bi[a#], Yihong Wang[a#], Heguo Yu[b], Xiaoyan Qian[a], Honghai Wang[a], Jun Liu[a,c*], Xuelian Zhang[a*]

[#] J.B. and Y.W. contributed equally to this work.

*Address correspondence to Xuelian Zhang (xuelianzhang@fudan.edu.cn) or Jun Liu (jun.liu@utoronto.ca).

Supplementary Information

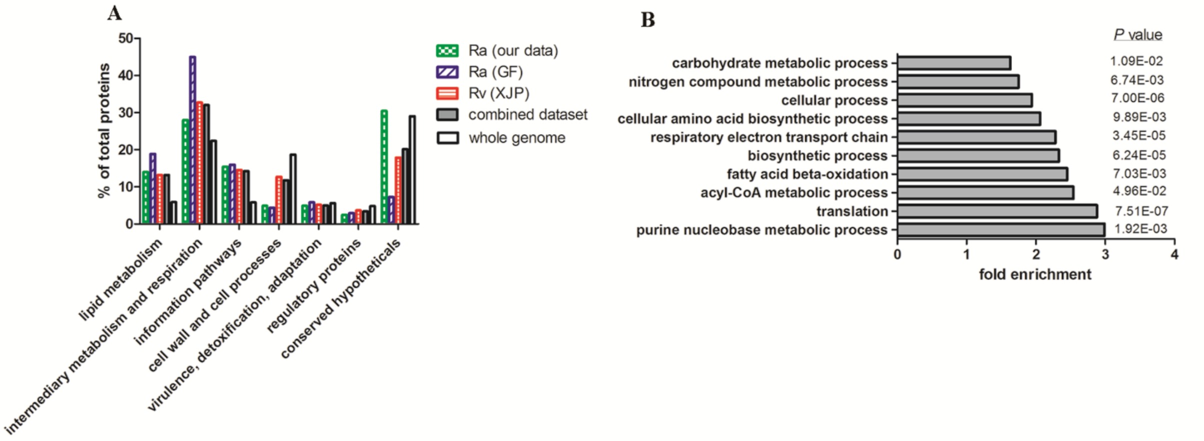

Fig S1. Functional distribution of acetylated proteins. **(A) Functional categories of acetylated *M. tb* proteins identified from three independent studies**. The portion of acetylated proteins in each functional group was plotted and compared with the distribution of functional groups in the whole genome. **(B) GO analysis of the 765 acetylated proteins identified from three independent studies**. Fold enrichment of biological processes that are significantly enriched (p<0.05) were plotted.

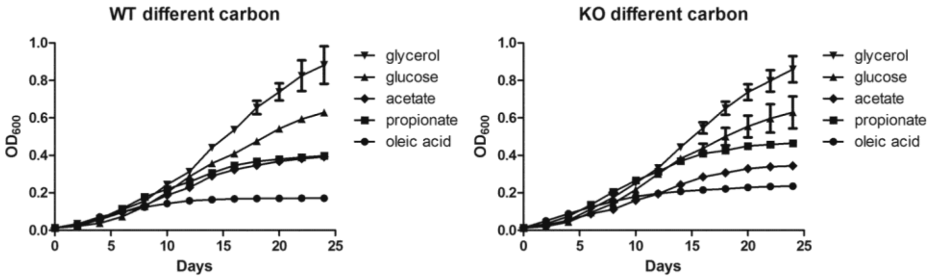

Fig S2. Growth of *M. tb* H37Ra WT and Δ*npdA* (KO) strains at different carbon source. Bacterial strains were grown in 7H9-10%OADC-0.05% tween80 to mid-log phase (OD_{600}=0.4-0.6), washed and resuspended in 7H9-0.05% tyloxapol. Aliquots (OD_{600}=0.5) of the cultures were diluted 1:50 (v/v) into 100 ml 7H9-0.5% BSA-0.085% NaCl-0.05% tyloxapol supplemented with individual carbon sources (5 mM of each): glucose, glycerol, sodium acetate, sodium propionate or oleic acids. The cultures were grown at 37°C for 24 days. Results are combined data from two independent experiments (mean ± s.d.), and each experiment was done in triplicate.

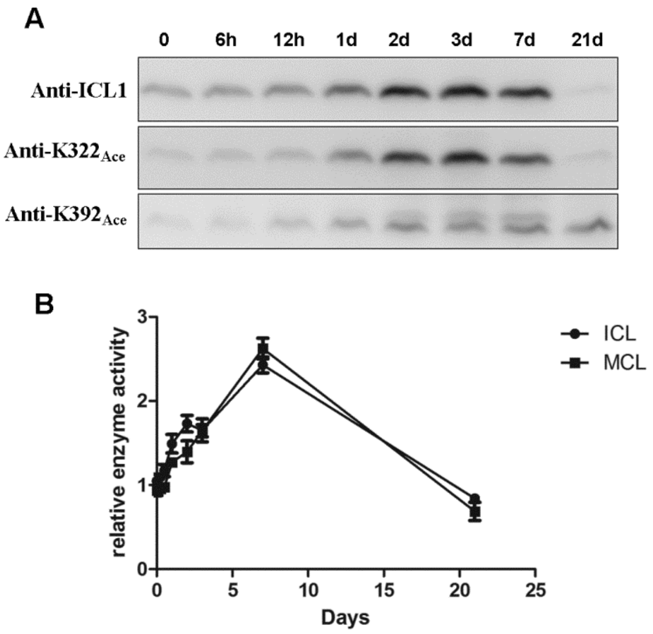

Fig S3. Expression and acetylation of ICL1 in Δ*npdA* cultures at different growth phase. (**A**) **Expression and acetylation of ICL1 at different growth time.** Δ*npdA* stain of *M. tb* H37Ra were grown in 7H9 media supplemented with 0.5% BSA, 0.085% NaCl and 5 mM propionate for 21 days. Cell extracts of cultures collected at indicated time points were prepared and analyzed by Western blot. Results are representative of three independent experiments. (**B**) **Enzymatic activities of ICL1**. Isocitrate lyase and methylcitrate lyase activities from Δ*npdA* culture grown in 5 mM propionate at different time points were analyzed. The data are normalized against the activity of Δ*npdA* at time 0 and are plotted as mean±s.d. (*n*=3).

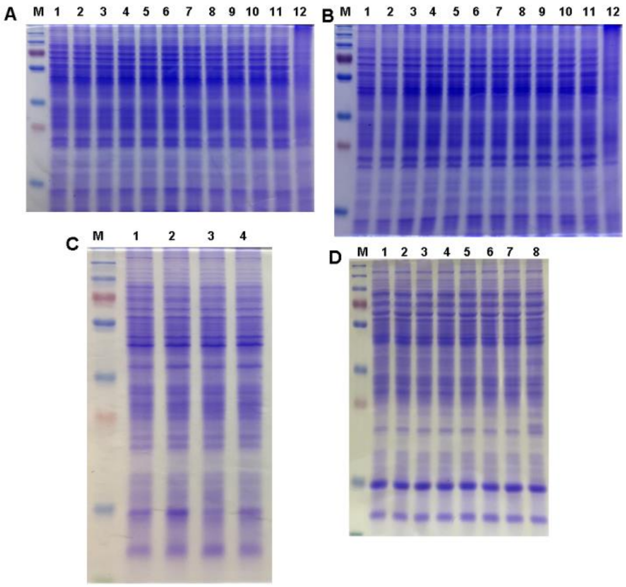

Fig S4. Loading control for Western blot analysis. *M. tb* cultures were grown at indicated conditions and cell extracts were prepared and equal amount (20 μg/lane) were analyzed by SDS-PAGE and stained with Coomassie blue. **(A & B) loading control for Fig 4B. (A)** WT cultures grown in indicated carbon sources. M: marker; lane 1: 0.05 mM glucose; lane 2: 0.5 mM glucose; lane 3: 5 mM glucose; lane 4: 0.05 mM acetate; lane 5: 0.5 mM acetate; lane 6: 5 mM acetate; lane 7: 0.05 mM propionate; lane 8: 0.5 mM propionate; lane 9: 5 mM propionate; lane 10: 0.05 mM oleate; lane 11: 0.5 mM oleate; lane 12: 5 mM oleate. (**B**) Δ*npdA* cultures grown in indicated carbon sources. M: marker; lane 1: 0.05 mM glucose; lane 2: 0.5 mM glucose; lane 3: 5 mM glucose; lane 4: 0.05 mM acetate; lane 5: 0.5 mM acetate; lane 6: 5 mM acetate; lane 7: 0.05 mM propionate; lane 8: 0.5 mM propionate; lane 9: 5 mM propionate; lane 10: 0.05 mM oleate; lane 11: 0.5 mM oleate; lane 12: 5 mM oleate. (**C**) **Loading control for Fig 5A.** M: marker; lane

1: WT culture grown to day 10; lane 2: WT culture grown to day 55; lane 3: Δ*npdA* culture grown to day 10; lane 4: Δ*npdA* culture grown to day 55. (**D**) **Loading control for Fig 5B.** WT cultures grown in 5 mM propionate to different time points. M: marker; lane 1: 0 hour; lane 2: 6 hour; lane 3: 12 hour; lane 4: 1 day; lane 5: 2 days; lane 6: 3 days; lane 7: 7 days; lane 8: 21 days.

Table S1. Growth phenotypes of Δ*npdA* using the BIOLOG Phenotype Microarray™ plates.

**Conditions**	**log_2 (Δ*npdA*/WT)**
pH 4.5 +L- Asparatic acid	3.69
D-Arabitol	3.07
Thymidine	2.90
Tween 40	2.03
Inosine	1.92
pH 5	1.80
pH 4.5+ Urea	1.70
pH 4.5 + L-Homoserine	1.69
pH 5.5	1.59
Glycogen	1.54
M-Tartaric Acid	1.53
L-Glutamine	1.40
β-Methyl-D-Galactoside	1.39
Glycine	1.36
β-D-Allose	1.31
L-Aspartic Acid	1.31
X-α- D-Glucuronide	1.28
Uridine	1.03
Mono Methyl Succinate	1.01
pH 6	1.00
L-Lyxose	-2.67
D-Xylose	-2.41
D,L-Lactamide	-2.30
L-Arabitol	-2.21
Cefazolin	-1.74
Salicin	-1.71
D-Gluconic acid	-1.44
γ -Amino Butyric Acid	-1.21
Malonic Acid	-1.16
L-Galactonic Acid -γ -L actone	-1.10
L-Asparagine	-1.07
